# Dry Fish Image dataset: Data-driven analysis and deep learning-based classification

**DOI:** 10.1016/j.dib.2026.112683

**Published:** 2026-03-13

**Authors:** Amran Hossain, Md Jakir Hossain, Iffat Ara Arin, Md Jisan Mia, Abir Hasan, Tasnia Tasnim Rupoma, Md Nawab Yousuf Ali

**Affiliations:** Department of Computer Science and Engineering, East West University, Dhaka, Bangladesh

**Keywords:** Image classification, Computer vision, Deep learning, Food recognition, Visual inspection, Machine learning applications

## Abstract

This article presents a dry fish image dataset framework to support data-driven research in computer vision, machine learning and Deep Learning. This dataset collected from a multiple market of dry fish in Dhaka, which shows that how different they look, how they are handled, and how they are presented in real world. Images were acquired using consumer-grade mobile cameras under natural lighting conditions to reflect practical deployment scenarios. The collection process purposefully incorporated variations in lighting, background clutter, camera angles, distances, and obstructions to augment data diversity. We got permission to take all the pictures and put them in a consistent naming and folder structure. The dataset has high-quality RGB images of twelve different types of dry fish, such as Bashpata, Chanda, Chapila, Chewa, Churi, Loitta, Shukna Feuwa, Shundori, Chingri, Kachki, Narkeli, and Puti Chepa. Each class includes dry fish species that are commonly traded and shows natural differences in size, texture, color, and drying patterns within the class. We looked at in the data and put them in groups by hand to make sure they were all in the same class by taking help of expert. We did some simple preprocessing, such as getting rid of duplicates and making sure that the formats were the same, all while keeping the data's original visual features.You can use this dry fish dataset again for things like classifying images, extracting features, analyzing data imbalance, benchmarking data augmentation, and visualizing explainable artificial intelligence. It could also help with research on how to recognize food with few resources, automate markets, digitize supply chains, and use mobile devices for inspections. The way the dataset is set up makes it easy to work with well-known deep learning frameworks. It can also be added to with more classes or metadata, making it useful for both academic research and practical development in smart food systems and fisheries informatics.

Specifications Table

**Table:** 

Subject	Computer Sciences
Specific subject area	Computer Vision and Image Processing
Type of data	Images
Data collection	Data were collected from People who sell dried fish often do so in local markets. Smartphone cameras took the pictures in natural light without any extra setup. Pictures were taken from different angles and distances to feel in real life. The backgrounds were not changed so that the natural visual context would stay the same. After the images were collected, they were checked for duplicates and low-quality files. Then, they were put into folders based on the different types of dry fish.
Total dataset size and format	Total 1.11 GB images and All images are stored in JPG format with RGB colour encoding
Data source location	Karwan Bazar Fish Market Dhaka, Bangladesh. Latitude: 23.754253° N Longitude: 90.393425° E
Data accessibility	Repository name: Dry Fish Image Dataset Data identification number: 10.17632/f3sr9xr8md.1 Direct URL to data: https://data.mendeley.com/datasets/f3sr9xr8md/1
Related research article	

## 1 Value of the Data

• Improving Research on Dry Fish Classification: These data represent a full set of images of 12 different categories of dry fish, including the natural variation of color, texture, and size. This variety is useful in creating, benchmarking and advancing computer vision models in food science, aquaculture, and quality inspection areas [1].
• Supporting the deep Learning Model Development: The dataset can be re-used by researchers to train, validate, and test deep learning models to solve image classification, object detection, or a segmentation problem. Its hierarchical labeling allows reproduction and evaluation of experiments and model behavior of various architectures [[2], [3], [4]].
• Enabling Cross-Disciplinary Studies: In addition to computer vision, the data can assist interdisciplinary studies in nutrition, fisheries management and supply chain monitoring by supplying visual reference data on species identification, quality and automated inspection systems.
• Inclusion of Algorithm Robustness Testing: The variations in the dataset such as lighting, background and fish orientation would make it a good test of both robustness and generalizability of image processing algorithms so that the models could be used in real world settings.

## 2 Background

The data set had been collected to give a prepared set of selected images of dry fish in 12 of the popular categories. The rationale is the necessity of standardized visual data that would be used to facilitate automated classification, quality evaluation, and monitoring in fisheries and food supply chains. Data available in this field are either small, disparate or lack accuracy of labeling and they limit the process of creating and testing computer vision algorithms to perform the tasks of identifying the species and conducting inspection activities. Photos were taken in diverse conditions in terms of different lighting, backgrounds, and orientation to portray real variations that are experienced in the marketplaces, storage facilities and processing centers. The dataset has been systematically arranged with class labels to be able to train, test and validate machine learning and deep learning models. On methodology, the data is statistically consistent with the computer vision research practice, and it includes processed, high-quality images that can be applied to the practice of supervised learning. In connection with the previously existing research on automated fish classification or quality detection, this dataset can improve reproducibility, enable cross-validation of models, and facilitate additional experimentation with an algorithm without the need to collect extra data.

## 3 Data Description

**Fig. 1:**
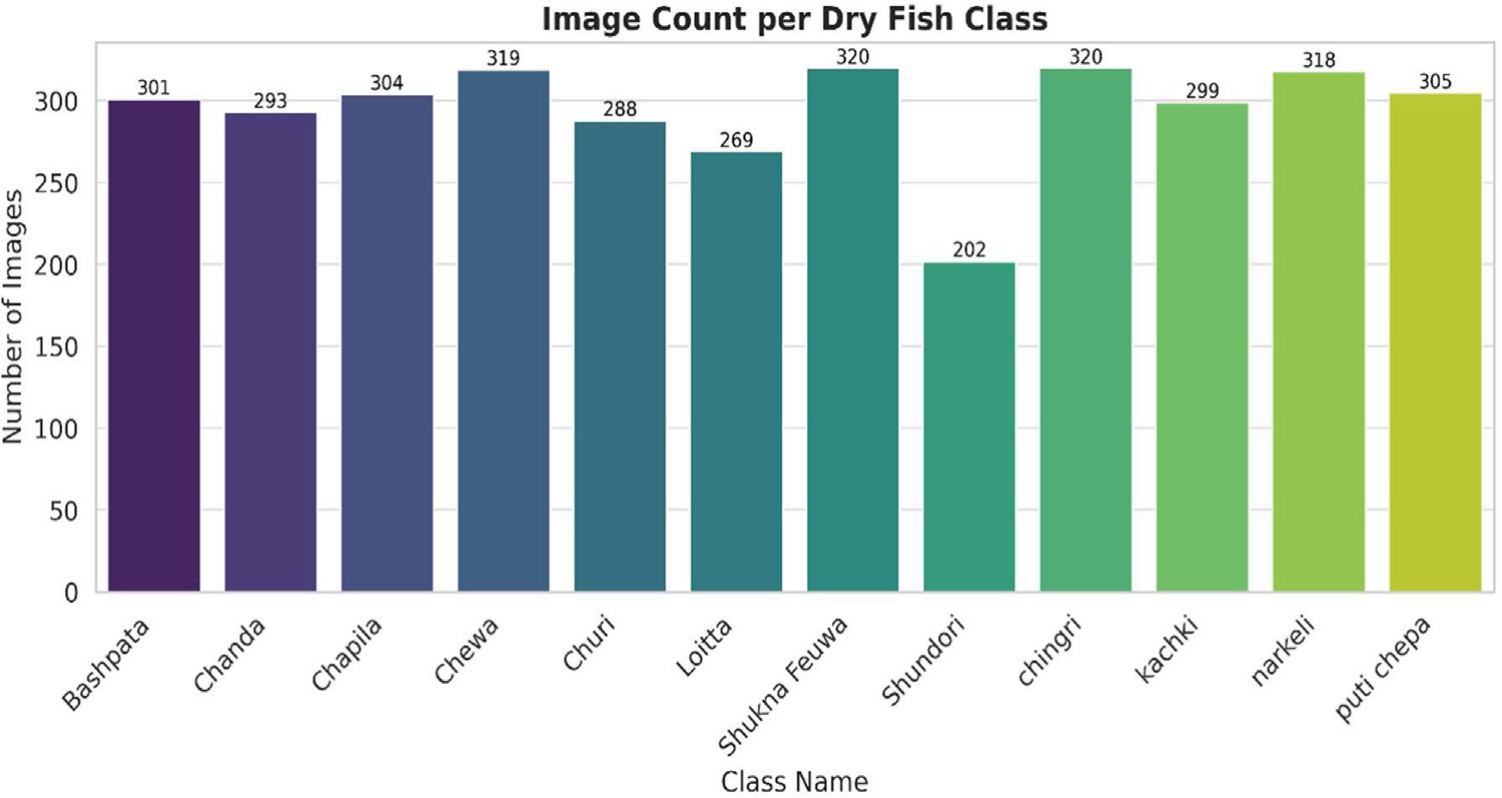
Class distribution of the Dry Fish Dataset.

**Fig. 2:**
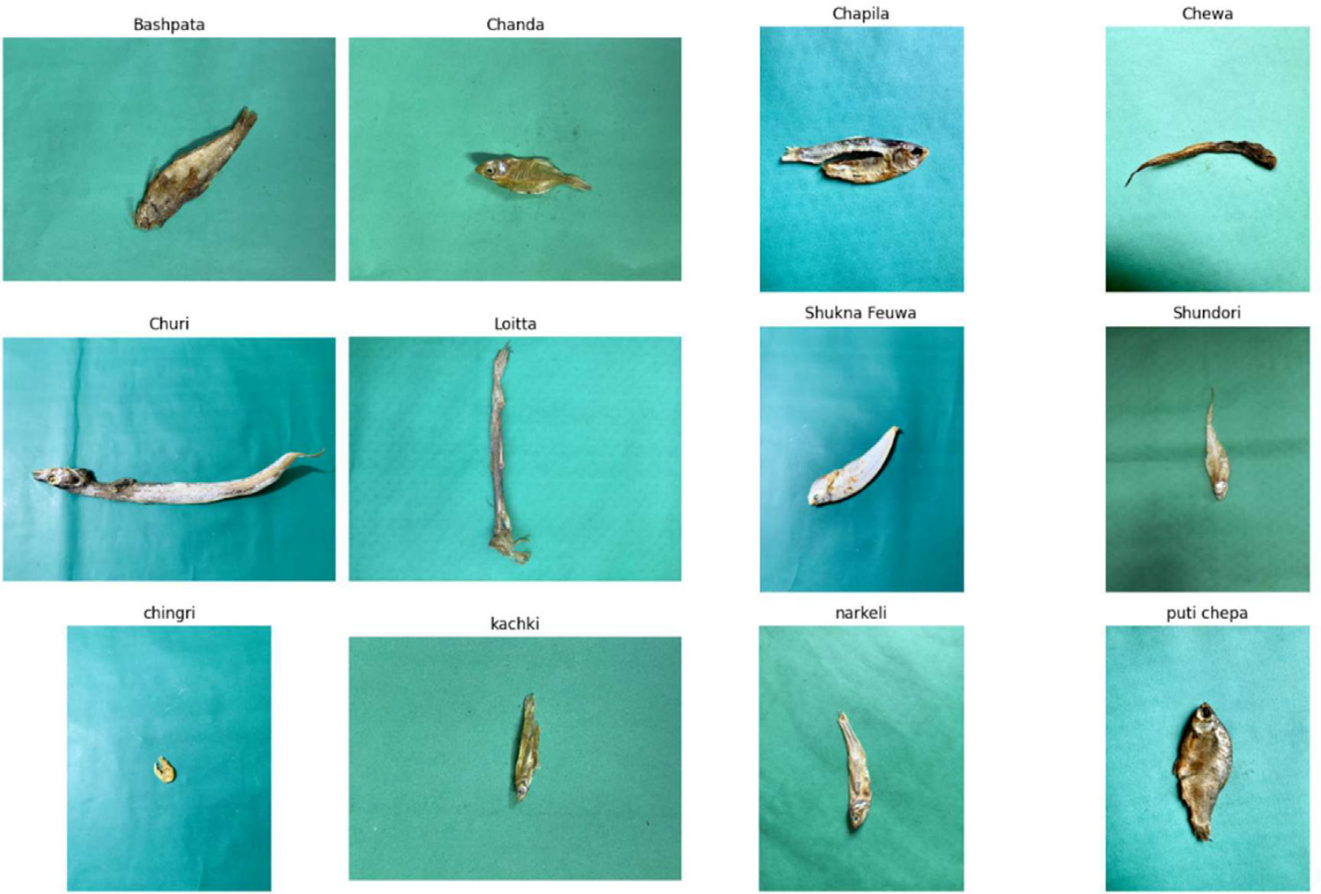
Representative samples from each class.

The collected dataset comprises twelve classes of dried fish, each representing a distinct species commonly found in local markets in Dhaka, Bangladesh. Dry Fish Image dataset has approximately 1.11 GB of storage, enabling to active researchers to plan computational resources. For each class, multiple samples were purchased and photographed under consistent conditions, as described in Section Dataset Collection. Species verification was performed with assistance from two local fisheries specialists who work in the dry fish market with >15 years of experience in fish trading and species identification in Dhaka markets. A total of 3533 images were acquired, with class distributions as follows in Table 1. Fig. 1 also shows the visualizations of distribution of images across the twelve dried fish classes. Fig. 2 Shows the Sample Images from each Dry Fish Class.

**Table 1:** Distribution and description of the twelve dried fish classes collected from local markets in Dhaka, Bangladesh.

Class Name	Number of Images	Description (Local Context)
**Bashpata**	301	Flat-bodied dried fish with elongated shape, typically sun-dried.
**Chanda**	293	Small, oval-shaped fish known for its translucent body texture.
**Chapila**	304	Medium-sized dried fish with silver scales, common in river catch.
**Chewa**	319	Slender-bodied fish, popular in coastal markets.
**Churi**	288	Long, ribbon-like fish with sharp head features.
**Loitta**	269	Cylindrical-bodied fish with distinctive dorsal fin pattern.
**Shukna Feuwa**	320	Curved-bodied dried fish, often heavily salted during processing.
**Shundori**	202	Small, slender fish with delicate body structure.
**Chingri**	320	Dried shrimp, small, used widely in curries and condiments.
**Kachki**	299	Small fish species with narrow bodies, usually sun-dried.
**Narkeli**	318	Medium-sized dried fish with firm body texture.
**Puti Chepa**	305	Flat-bodied dried fish with noticeable fin edges.

## 4 Experimental Design, Materials and Methods

### 4.1 Dataset acquisition

**Fig. 3:**
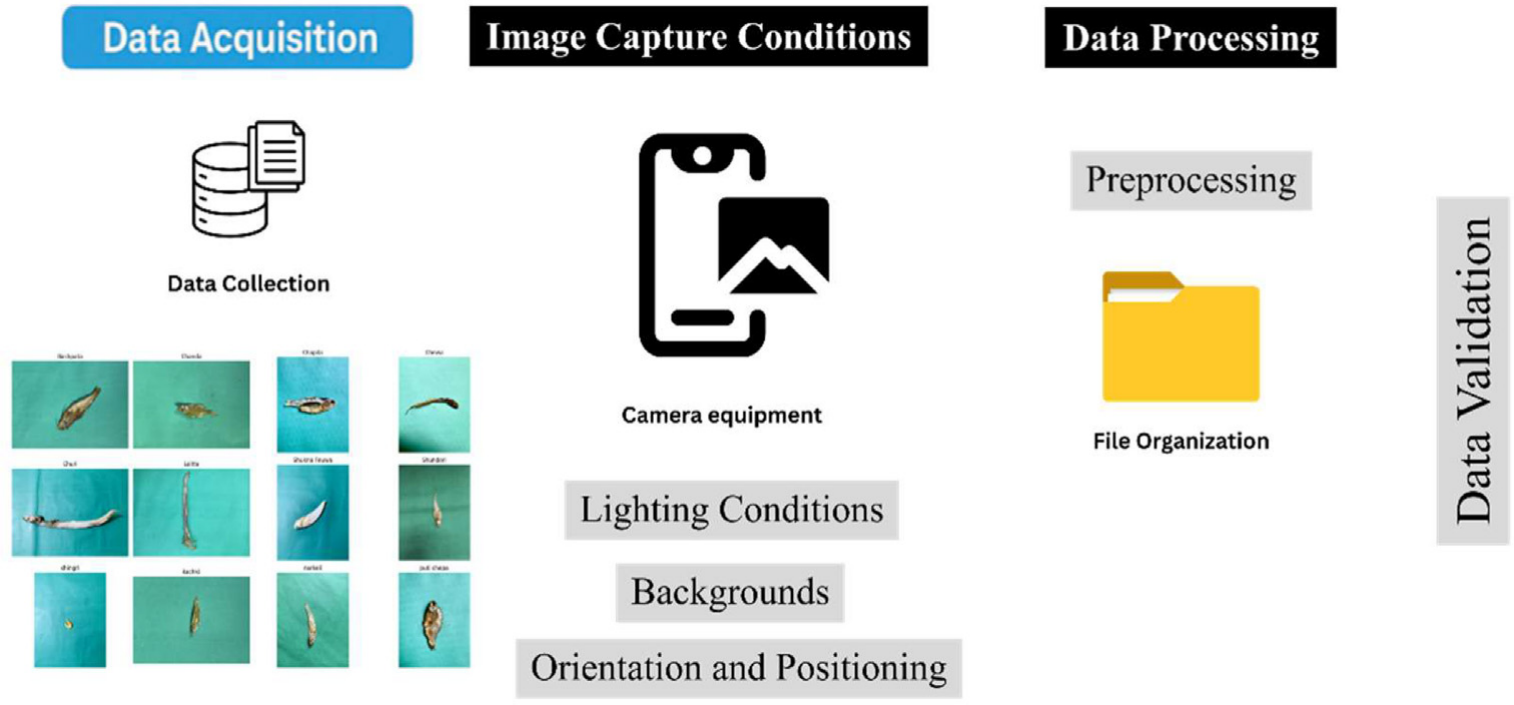
Dataset acquisition workflow.

It has twelve varieties of dry fish, that is, Bashpata, Chanda, Chapila, Chewa, Churi, Loitta, Shukna Feuwa, Shundori, Chingri, Kachki, Narkeli and Puti Chepa. To ensure that the size, shape, color, and texture of the fish were captured, 3533 high-resolution images were produced by looking at several sources, such as local markets, fisheries, and storage facilities. Every picture is assigned to a specimen of a fish, and it is also marked by its type of species. Fig. 3 shows the Data Acquisition process.

### 4.2 Image capture conditions

**Camera equipment** good quality cameras on smartphones (Iphone 13,One Plus nord) with a resolution of at least 12 Megapixels.

**Lighting Conditions:** Images were captured in natural daylight and the artificial lighting captures the changes in the illumination.

**Backgrounds:** Plain and Green backgrounds were applied to portray real life situations in markets and processing workplace conditions.

**Orientation and Positioning:** The fish specimens have been photographed at different angles such as, top, side view, and oblique views to document differences in their structure and textures.

### 4.3 Data processing

**Preprocessing:** Pictures were scaled to 224 × 224 pixels to fit the models. OpenCV and PIL libraries of Python were used to normalize and standardize pixel values.

**File Organization:** The images were sorted into directories with their respective labels in the classification to make learning through supervision easier. All images are stored in JPG format with RGB color encoding.

### 4.4 Software and code

**Programming Environment:** Python 3.10 with such libraries as torch, torch vision, numpy, pandas and opencv-python.

**Scripts:** Image resizing, normalization and labeling scripts were custom written. Every code is maintained through GitHub.

### 4.5 Data validation

Visual inspection of each image was done to check proper labeling and quality of the image. Motion blurred images, ones with markers that did not fit, and wrongly marked images were discarded.

### 4.6 Results of applying deep learning models

**Fig. 4:**
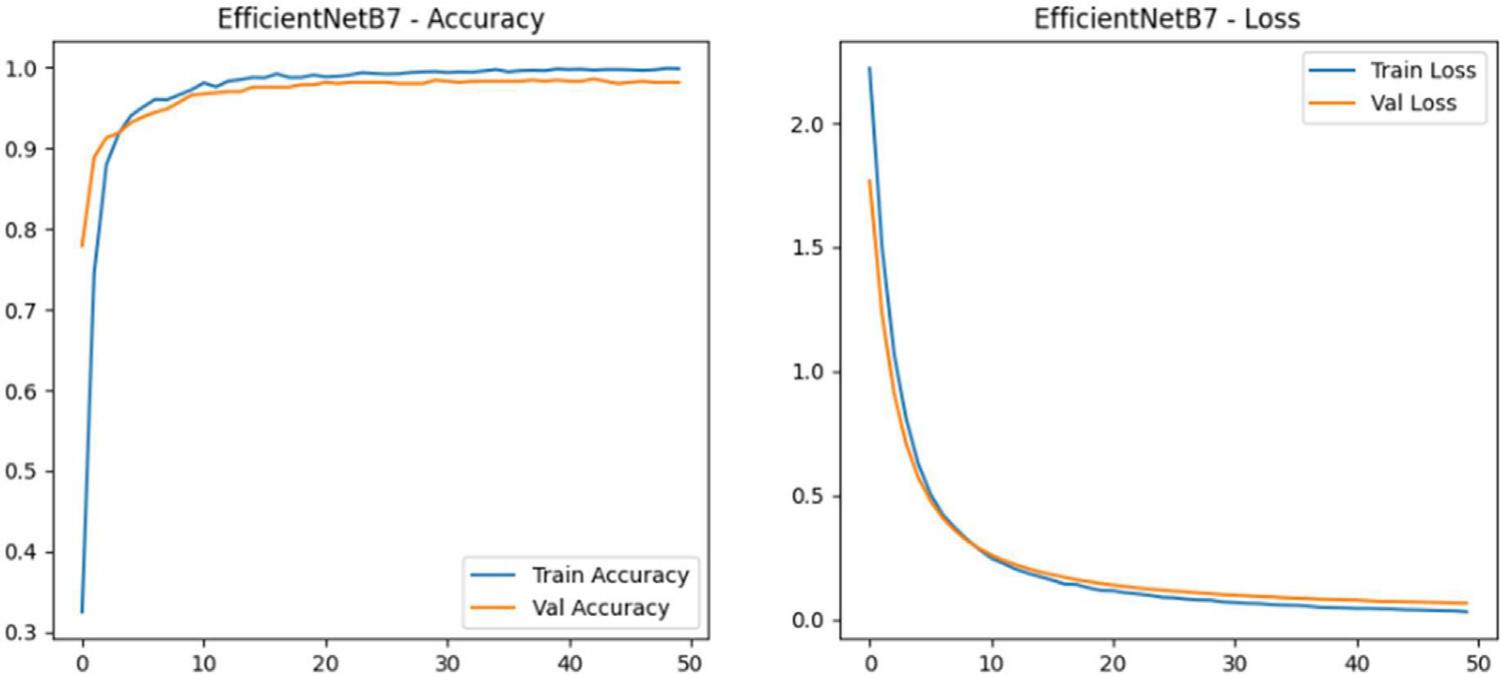
Performance of EfficientNetB7 (transfer learning) (accuracy vs loss).

**Fig. 5:**
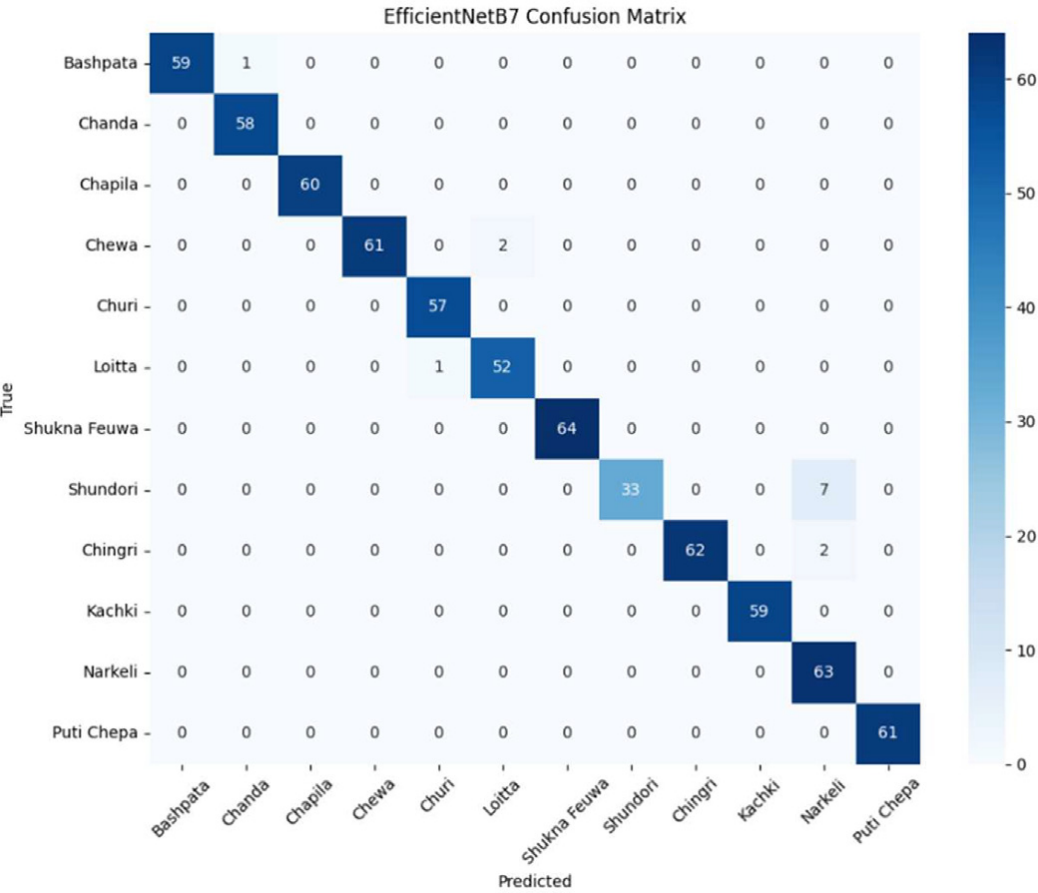
Performance of EfficientNetB7 (transfer learning) (confusion matrix).

**Fig. 6:**
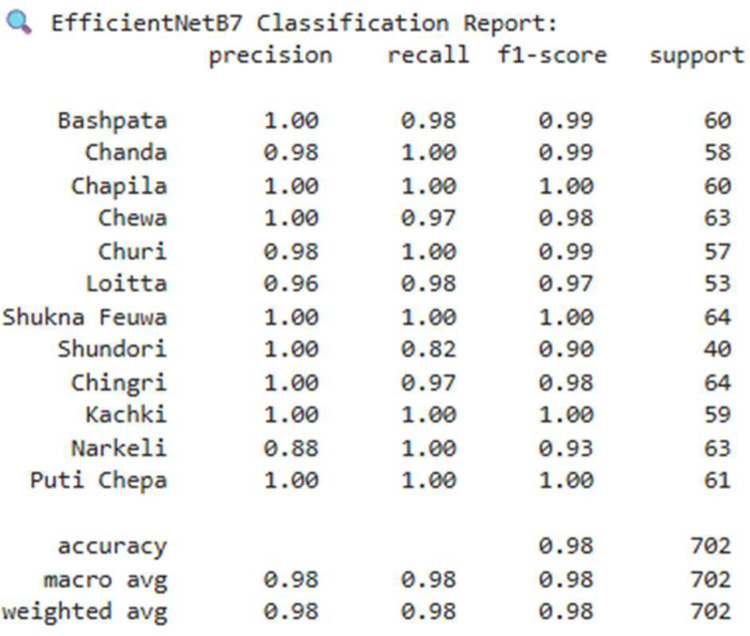
Classification report of EfficientNetB7 (transfer learning).

In this dataset, we used seven deep learning architectures on the Dry Fish Dataset to examine their performance in labelling several fish categories. The models entail custom-designed Convolutional neural networks (CNNs) as well as state of the art transfer learning techniques. Precisely, we assessed: Custom CNN Model 1,Transfer Learning using EfficientNetB7,Custom CNN Model 1 with L1 Regularization, Custom CNN Model 1 with L1 + L2 Regularization (ElasticNet), MobileNetV2,DenseNet121 In all datasets, each model went through the same experimental procedure, where the model is trained on the basis of categorical cross-entropy loss, Adam optimizer, and early stopping to avoid overfitting. Training and training (test) sets were evaluated, and performance in terms of accuracy and loss was scored. Table 2 shows the Performance comparison of deep learning models on the Dry Fish Dataset. Fig. 4, Fig. 5, Fig. 6 shows the EfficientNetB7 performance evolution.

**Table 2:** Performance comparison of deep learning models on the Dry Fish Dataset.

Model	Training Accuracy	Validation Accuracy	Training Loss	Validation Loss
Custom CNN Model 1	75.115 %	92.88 %	0.6009	0.2652
**EfficientNetB7 (Transfer Learning)**	**99.69%**	**98.15%**	**0.0337**	**0.0658**
Custom CNN 1 + L1 Regularization	88.10 %	93.16 %	1.4117	1.3767
Custom CNN 1 + L1+L2 (ElasticNet)	81.43 %	89.89 %	0.9601	0.8409
MobileNetV2	99.11 %	96.30 %	0.0561	0.1203
DenseNet121	94.51 %	94.30 %	0.1684	0.1799

The results of the experiment bring out tradeoffs in accuracy of classification and computational efficiency of dried fish classification. EfficientNetB7, which applies transfer learning, had the best accuracy (98.15 % validation) and but required too much memory and time to inquire, making it unsuitable for resource-constrained scenarios. Lightweight models such as MobileNetV2 and Custom CNN 2 have a shorter inference time and use less memory and are suitable on edge devices but a bit less accurate. Grad-CAM visualizations ensured that models emphasize significant features that include shape, texture and pattern which increase interpretability. An element combination of ResNet50, EfficientNetB0, MobileNetV2, and DenseNet121 had an overall accuracy of 99.98 to both the training and the validation set, which is more robust and generalizes better than the single models.

## Limitations

The dataset, though diverse, may not capture all variations in fish species, drying methods, or environmental conditions, which can limit model generalization. Shundori, Churi, Loitta class have fewer photos than others, but no targeted augmentation was used, therefore the model's resilience could be tested with a small class imbalance.

## Ethics Statement

Our Research confirm that this work does not involve human subjects, animal experiments, or data collected from social media platforms. All ethical requirements for publication in *Data in Brief* have been followed.

## Credit Author Statement

**Amran Hossain:** Data collection, Data curation, Writing. **Md Jakir Hossain:** Conceptualization, Data collection, Model Development, Writing – Review & Editing. **Iffat Ara Arin:** Investigation, Data curation, Visualization. **Md Jisan Mia**: Data collection, Validation, Experimentation. **Abir Hasan:** Resources, Data collection, Writing. **Tasnia Tasnim Rupoma:** Resources, Writing**. Md Nawab Yousuf Ali:** Supervision, Writing – Review & Editing

## Data Availability

Mendeley DataDry Fish Image Dataset (Original data).
